# Gallbladder Paraganglioma: A Mysterious Histological Finding

**DOI:** 10.7759/cureus.35327

**Published:** 2023-02-22

**Authors:** Jee Eun Do, Kathryn Harvey, Abdul Rana, Adam Swalling, Martin Bruening

**Affiliations:** 1 Department of Surgery, The Queen Elizabeth Hospital, Adelaide, AUS; 2 Department of General Surgery, Lyell McEwin Hospital, Adelaide, AUS; 3 Department of Pathology, The Queen Elizabeth Hospital, Adelaide, AUS

**Keywords:** nonfunctional paraganglioma, cholecystectomy, gallbladder paraganglioma, paraganglioma, extra-adrenal paraganglioma

## Abstract

Gallbladder paragangliomas are extremely rare with only a handful of cases reported so far. There are no definitive guidelines for the management of gallbladder paragangliomas due to their rarity. We present a case of a 53-year-old male who was found to have gallbladder paraganglioma post-laparoscopic cholecystectomy, performed for right upper abdominal pain. On review of the literature, all previously reported cases had been nonsecretory and benign. For patients who have no symptoms of secretory paragangliomas and no family history of endocrine syndromes, cholecystectomy and clinical follow-up may be a sufficient initial management following an incidental finding of gallbladder paraganglioma.

## Introduction

Cholecystectomy is a commonly performed operation, especially in the Western population, for indications relating to cholelithiasis, such as biliary colic and acute cholecystitis [1]. About 10%-15% of the adult Western population will develop gallstones in their lifetime, and cholecystectomy is the mainstay of treatment [1]. A routine postoperative histological examination is performed on the removed gallbladder specimen to rule out malignancy as the potential precipitant of pain and inflammation [1]. Gallbladder paraganglioma is certainly an unexpected histological finding following cholecystectomy, as it is very rare with only 24 case reports found in the literature to date. Owing to its rarity, no specific guidelines exist on management, further investigations, and surveillance. This article reviewed 21 out of 24 reported cases of gallbladder paragangliomas in the current English literature to evaluate the consensus for further investigations and surveillance in cases of incidental gallbladder paraganglioma following cholecystectomy.

## Case presentation

A 53-year-old male presented with an eight-day history of right upper quadrant (RUQ) abdominal pain, which started suddenly and persisted. His vital signs were all within normal ranges (blood pressure of 130/92, heart rate of 79, and temperature of 36.2°C). Abdominal examination was unremarkable with negative Murphy’s sign. Laboratory tests including complete blood examination, C-reactive protein, and lipase were all normal, deviating away from differentials such as acute cholecystitis and gallstone pancreatitis. Liver function tests were mildly deranged, with alkaline phosphatase of 183 U/L, gamma-glutamyl transferase of 237 U/L, alanine aminotransferase of 148 U/L, and aspartate aminotransferase of 115 U/L. Abdominal ultrasonography (US) showed mild hyperemia along the gallbladder wall with mobile sludge, but no other features of acute cholecystitis such as pericholecystic fluid.

He was admitted for pain control and monitoring, and due to persistent RUQ pain, he was taken for laparoscopic cholecystectomy two days after presentation. Intraoperatively, the gallbladder appeared normal with only mild wall edema. His blood pressure was stable between 110 and 130 mmHg (systolic) throughout the operation and admission. He was discharged with complete resolution of pain and no perioperative complications.

**Figure 1:**
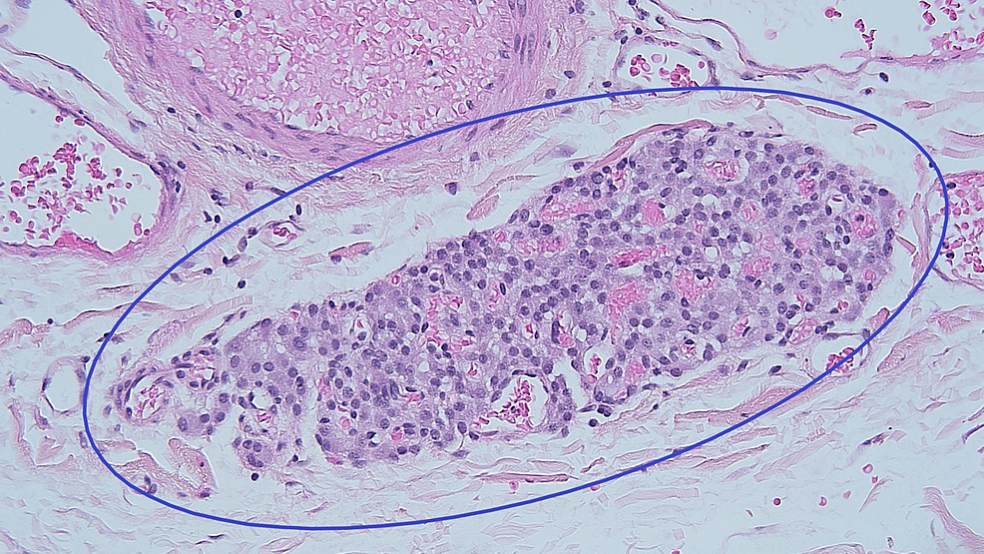
Paraganglioma with hematoxylin and eosin stain (magnification: ×200) 0.5-mm focus of paraganglioma centered in the adventitial adipose tissue

**Figure 2:**
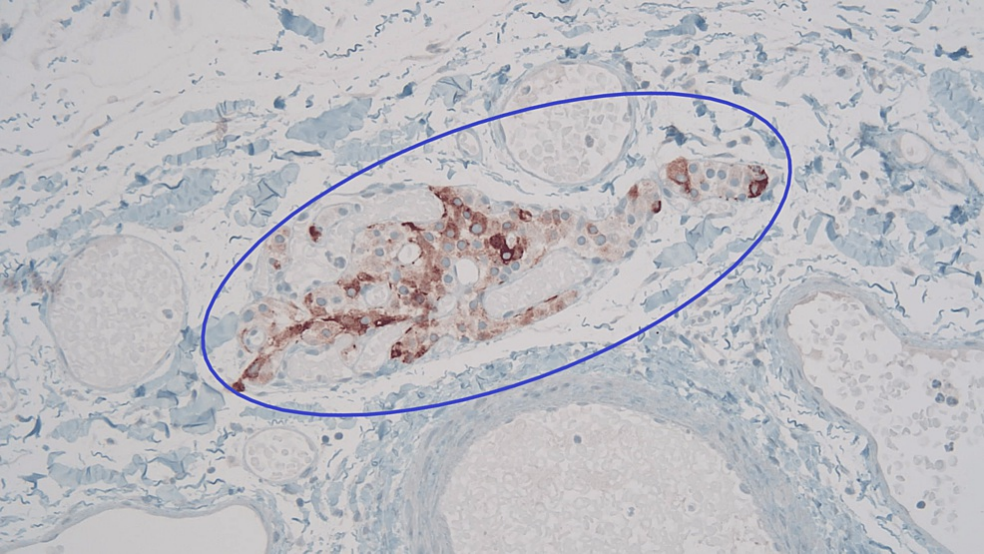
Paraganglioma with chromogranin A stain (magnification: ×100) Tumor expressing strong labeling with the neuroendocrine marker chromogranin A

**Figure 3:**
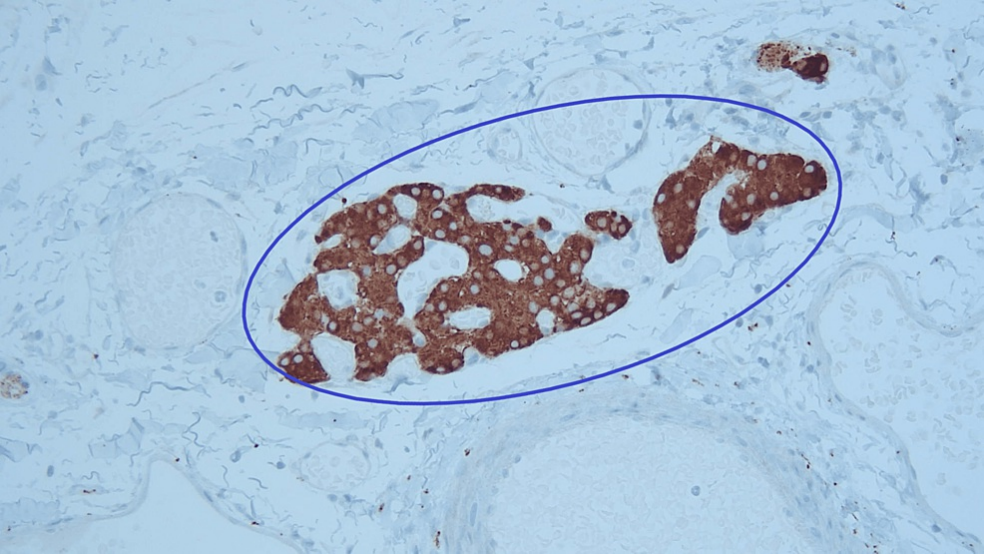
Paraganglioma with synaptophysin stain (magnification: ×100) Tumor expressing strong labeling with the neuroendocrine marker synaptophysin

**Figure 4:**
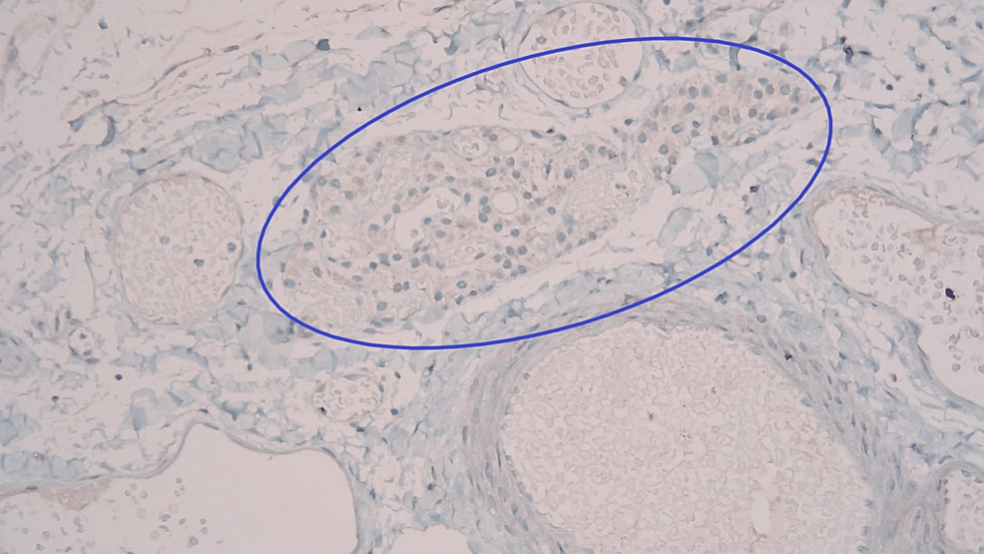
Paraganglioma with cytokeratin AE1/3 stain (magnification: ×100) Tumor expressing negativity for the cytokeratin AE1/3, militating against carcinoma

Macroscopically, the gallbladder contained no gallstones and had yellow mucosal flecks, which raised suspicion for cholesterolosis. The wall had a normal thickness of 2 mm. Microscopic examination revealed a single, 0.5-mm tumor centered in the adventitial adipose tissue, which consisted of a well-circumscribed aggregate of tightly packed epithelioid cells (Figure 1). The lesional cells were uniform in appearance, and there were no concerning histological features such as mitotic activity, invasion, or high-grade cytomorphology. This lesion showed strong labeling with the neuroendocrine markers synaptophysin and chromogranin A (Figure 2 and Figure 3). Negativity for the cytokeratin AE1/3 militated against carcinoma (Figure 4). The combined morphological and immunohistochemical features were those of paraganglioma. The lesion was well clear of the cystic duct margin. There were no other foci of paraganglioma identified in the remaining gallbladder. There were background changes of chronic cholecystitis with underlying changes of cholelithiasis and cholesterolosis.

## Discussion

Paragangliomas are neoplasms of the paraganglia, originating from the neural crest cells [2,3]. Paragangliomas can be categorized based on location, adrenal (also known as pheochromocytoma), and extra-adrenal. The typical locations for extra-adrenal paragangliomas include the sympathetic plexus in the preaortic and paravertebral regions and the skull base such as around the jugular foramen, ears, and carotid bodies [2,3]. They can also be categorized as parasympathetic or sympathetic; parasympathetic paragangliomas are usually asymptomatic and nonsecretory, with less than 5% secreting catecholamines [2,3]. In contrast, sympathetic paragangliomas mainly secrete noradrenaline and cause symptoms similar to those caused by pheochromocytoma, such as palpitations and sweating [3]. Most cases of paragangliomas are sporadic; however, 30%-40% of cases are familial and occur as a manifestation of genetic syndromes, most commonly, multiple endocrine neoplasia (MEN) types 2A and 2B [3].

An online search using the keywords “gallbladder,” “paraganglia,” and “paraganglioma” on the National Library of Medicine found 24 cases of gallbladder paragangliomas since 1972 (Table 1). These included two case reports from 1990, which were only available as abstracts, and one in Spanish; these were excluded from the review. Most cases had been found incidentally after cholecystectomy for indications such as cholelithiasis and abdominal pain. In three cases, abnormal appearance of the gallbladder was detected on imaging performed for symptoms not related to the gallbladder (one computed tomography (CT) of the chest and two positron emission tomography (PET)) [2,4,5]. Two out of these three cases underwent cholecystectomy, confirming the presence of gallbladder paraganglioma histologically [4,6]. Interestingly, the two cases that were found on the PET scan were associated with synchronous pancreatic neuroendocrine tumor and other paragangliomas [4,5]. One case report by Mehra and Chung-Park of a 36-year-old male who underwent a Roux-en-Y gastric bypass procedure for morbid obesity turned out to have a family history of pheochromocytoma in one of the patient’s siblings on retrospective follow-up, but no other family history concerning for endocrine syndromes [7].

**Table 1:** Summary of reported cases of gallbladder paraganglioma from 1972 to the present, arranged in chronological order M: male, F: female, NM: not mentioned, RUQ: right upper quadrant, CT: computed tomography, PET: positron emission tomography

Case	Source	Year reported	Age (year), sex	Clinical presentation	Size (cm)	Management
1	Miller et al. [8]	1972	57, M	Recurrent hematemesis	3	Cholecystectomy
2	Wolff [9]	1973	32, F	Cholelithiasis	NM	Cholecystectomy
3	Wolff [9]	1973	52, F	Cholelithiasis	NM	Cholecystectomy
4	Wolff [9]	1973	53, F	Cholelithiasis	NM	Cholecystectomy
5	Kawabata [10]	1999	51, F	Mildly elevated transaminases	NM	Cholecystectomy
6	Kawabata [10]	1999	55, F	Epigastric pain	NM	Cholecystectomy
7	Hirano [11]	2000	58, F	Right hypochondrial pain	1.3 × 0.9	Cholecystectomy
8	Cho et al. [12]	2001	45, F	RUQ pain	2.5	Cholecystectomy
9	Mehra et al. [7]	2005	36, M	Asymptomatic, incidental finding following cholecystectomy done during Roux-en-Y bypass	1.5	Cholecystectomy
10	Ece et al. [13]	2014	57, F	RUQ pain	1.8	Cholecystectomy
11	Koplay et al. [14]	2014	57, M	RUQ pain	2.5	Cholecystectomy
12	AlMarzooqi et al. [15]	2018	NM	RUQ pain	0.25	Cholecystectomy
13	Sater et al. [6]	2019	36, M	Mild hypertension, tinnitus, found to have multifocal paragangliomas, hyper-enhancing gallbladder lesion on MRI of the abdomen, further investigation with DOTATATE PET showed intense avidity	2.1	Cholecystectomy
14	Mahin et al. [2]	2019	79, M	Asymptomatic, incidental gallbladder wall mass found on CT of the chest	3 × 3	Cholecystectomy
15	Corten et al. [16]	2019	27, F	RUQ pain	0.3	Cholecystectomy
16	D'John et al. [17]	2020	63, F	RUQ pain	<1	Cholecystectomy
17	Aaquist et al. [4]	2020	74, M	Asymptomatic, incidental finding on DOTATATE PET scan performed for investigation of pulmonary infiltrates, synchronous pancreatic neuroendocrine tumor found	2.2 × 1.6 × 1.1	Cholecystectomy
18	Song et al. [18]	2021	48, F	Abdominal pain	1.6	Cholecystectomy
19	Cho et al. [19]	2021	48, F	Abdominal pain	1	Cholecystectomy
20	Shreya et al. [5]	2021	72, F	Bilateral otorrhea, reduced hearing, CT and DOTATATE PET, soft tissue mass in the left hypotympanum and bilateral carotid spaces and gallbladder mass	NM	No intervention
21	Oztas et al. [20]	2022	61, F	NM	2	Cholecystectomy
22	Present case	2022	53, M	RUQ pain	0.5	Cholecystectomy

Radiologically, paragangliomas do not exhibit any specific features on CT or magnetic resonance imaging (MRI). Extra-adrenal paragangliomas may show a similar appearance to a hemangioma on a CT due to its hypervascularity, and on T2-weighted MRI, it may exhibit a “salt and pepper” pattern over the regions with high and low signal intensities [20,21]. The case report by Mahin et al. showed that gallbladder paraganglioma was intensively hyper-enhancing in the arterial phase on CT, raising concern for malignancy, which prompted cholecystectomy [2]. Abdul Sater et al., who reported a case of a 38-year-old male with succinate dehydrogenase subunit D (*SDHD*) gene mutation, suggested Gallium-DOTATATE positron emission tomography (PET) as an essential follow-up investigation for any patients with hereditary paragangliomas, as the gallbladder paraganglioma in this male lacked F-fluorodeoxyglucose (F-FDG) avidity but was intensely avid with Gallium-DOTATATE [6].

There are no guidelines for the management and surveillance of incidental gallbladder paragangliomas due to their rarity. On review of the literature, there are no reports of complicated postoperative recovery. The authors of a case report of hemorrhagic gallbladder paraganglioma repeated abdominal CT at six weeks postoperatively, which did not show any evidence of tumor recurrence or any significant findings; however, they did not comment on whether the CT was performed to ensure that there was no further hemorrhage or for surveillance for paraganglioma [18]. Blood and urine catecholamine may be considered to look for metastatic or residual disease [3]. However, this would not yield any result in patients with nonsecretory tumors, which, so far, all reported cases had been. There are no reports of association with MEN syndrome or malignancy.

Our patient was informed of the diagnosis, and the case was discussed in a multidisciplinary meeting. As he did not have any other symptoms and no family history of endocrine syndromes, the decision for no further investigation was made.

## Conclusions

The majority of gallbladder paragangliomas were diagnosed incidentally after cholecystectomy for abdominal pain. They were benign, nonsecretory tumors occurring in patients with no known familial multiple endocrine syndromes. Blood and urine catecholamine levels are not likely to be of any diagnostic utility, and there are no specific abdominal US, CT, or MRI features to detect metastatic, recurrent, or residual tumors. There have been a few cases of gallbladder paragangliomas occurring with other extra-adrenal paragangliomas, and these were avid on Gallium-DOTATATE PET. For patients who have no symptoms of secretory paragangliomas and a history of familial endocrine syndromes, immediate investigation with further imaging may not be required, and clinical follow-up may be adequate. Patients who have or develop symptoms suggestive of other extra-adrenal paragangliomas or have a family history of endocrine syndromes may benefit from Gallium-DOTATATE PET.
